# Wandering Minds: Tracing Inner Worlds Through a Historical-Geographical Art Installation

**DOI:** 10.1080/2373566X.2018.1441736

**Published:** 2018-05-01

**Authors:** Hilary Powell, Hazel Morrison, Felicity Callard

**Affiliations:** ^a^ Durham University; ^b^ Birkbeck, University of London

**Keywords:** archive, art exhibition, audiovisual installation, interdisciplinary, mind wandering

## Abstract

The human act of wandering across landscapes and cityscapes has carved the research interests of scholars in cultural, urban, and historical geography, as well as in the humanities. Here we call for—and take the first steps toward—a historical geography of wandering that is pursued in the head rather than with the legs. We do so through analyzing how our audiovisual installation on mind wandering opened up epistemological and ontological questions facing historical geographies of the mind. This installation both modeled mind wandering as conceptualized at different historical moments and aimed to induce mental perambulation in its visitors. In so doing, it was intended both to stage and to disrupt relations between body and mind, the internal and external, attention and inattention, motion and stillness—and, importantly, between the archival and that which resists archival capture. We reflect on how we interspersed traditional scholarly historical and geographical enquiry with methods gleaned from creative practices. In particular, we consider the challenges that such practices pose for how we conceptualize archives—not least when the focus of attention comprises fugitive mental phenomena.

## Abstract (zh)

人类在地景与城市景观之间的漫游行动，开拓了文化、城市与历史地理学者以及人文学者的研究旨趣。我们于此呼吁在脑中进行、而非透过双脚进行的漫游历史地理学，并向该目标初步迈进。我们通过分析心灵漫游的视听设置，如何开启心灵历史地理学所面临的认识论与本体论问题。此一设置同时将心灵漫游模式化成为在不同历史时刻的概念化，并旨在引发拜访者的心灵巡迴。通过这麽做，本研究意图同时展现并颠覆身体与心灵、内在与外在、注意与无意、动态与静态之间的关系，以及重要的是，档案与抗拒档案取得之间的关系。我们反思自身如何以创意实践中拾获的方法，点缀传统历史与地理的学术探问。我们特别考量此般实践对于我们如何概念化档案所带来的挑战——特别是当关注焦点包含短暂易变的心灵现象之时。

## Abstract (es)

La acción humana de deambular a través de paisajes rurales y urbanos ha tallado los intereses investigativos de estudiosos en geografía cultural, urbana e histórica, lo mismo que en las humanidades. Aquí nosotros clamamos ––y damos los primeros pasos al respecto–– por una geografía histórica del deambular que se persiga más con la cabeza que con las piernas. Emprendemos esa tarea analizando cómo nuestra construcción visual sobre el deambular de la mente abrió cuestiones epistemológicas y ontológicas que enfrentan geografías históricas de la mente. Esta construcción a la vez modeló el deambular de la mente según se le conceptualice en diferentes momentos históricos y apuntó a inducir deambulación mental en sus visitantes. Al hacerlo, se intentó poner en escena y afectar las relaciones entre el cuerpo y la mente, lo interno y lo externo, la atención y la desatención, movimiento y quietud ––y, muy importante, entre lo archivístico y aquello que se resiste a la captura en archivo––. Reflexionamos sobre la manera como intercalamos la indagación docta histórica y geográfica tradicional con métodos derivados de prácticas creativas. Consideramos particularmente los retos que plantean tales prácticas sobre el modo como conceptualizamos los archivos ––menos aun cuando el foco de atención consta de fenómenos mentales huidizos.

## STEP INTO THE *CUBICULUM*

*You pause at the end of the deserted residential street. The buzz of traffic from the Mile End Road has dwindled to a distant hum. Across the car park you see the gallery, a low, unpretentious building standing in a spur of urban parkland. As you enter the exhibition space the glass doors fall shut behind you, sealing out all outside sound. Tall, windowless walls to your left and right curve inward, directing your gaze to the lake, the gallery’s external centerpiece, visible from every angle through a vast, convex glass wall. Oriented due west, the gallery and nearside bank of the lake are bathed in a warm, golden light. The further bank lies shrouded in shadow below the silhouetted skyline of east London. It is 4 p.m. A couple, holding hands, are watching something on a screen at the far end of the room while an older man sits on an upholstered bench looking out at the lake. The exposed pipework and hard surfaces that made the hall seem cacophonous on opening night now make it feel empty and echoey. The untreated medium-density fiber (MDF) boards and supports that divide up the space heighten this impression. A large MDF box stands in the center of the space. It bears the same unfinished aesthetic as the rest of the exhibits but unlike the surrounding screens it offers no indication of what it holds. Walking around the box, a pair of floor-length black curtains indicate, but continue to occlude, the entrance. You are reminded of going to have your passport photo taken as a child. Given the deserted feel of the gallery, you take a chance on it being unoccupied and poke your head through the curtains.*

**FIGURE 1:**
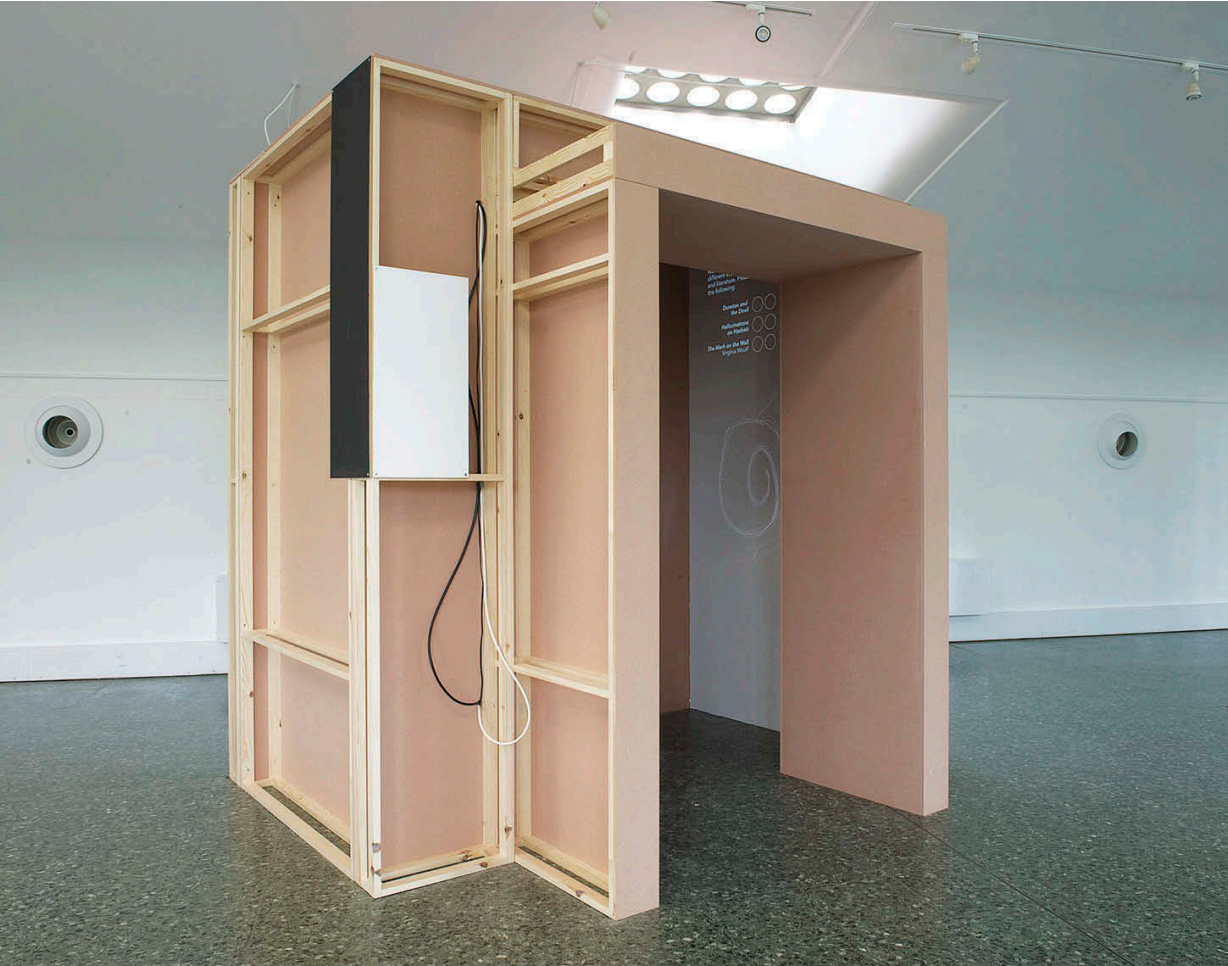
The *Cubiculum*—without curtains, so as to reveal the projection screen inside. Photo by Peter Kidd; used with permission. (Color figure available online.)

**FIGURE 2:**
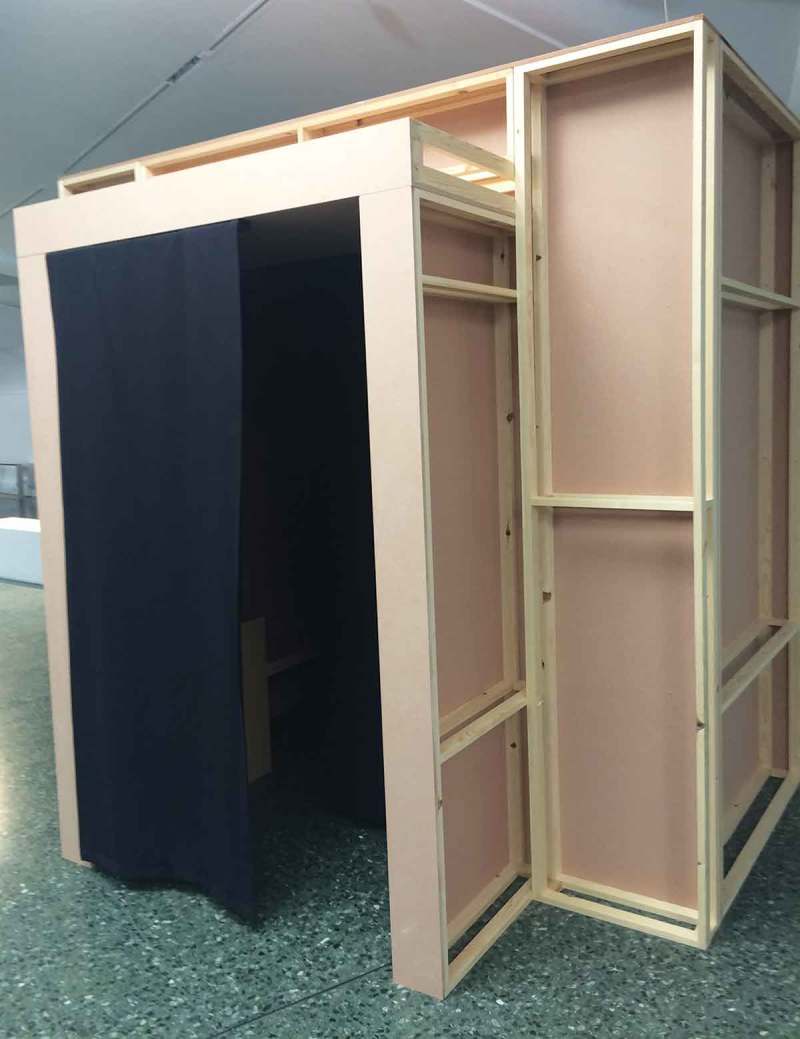
The *Cubiculum*—with curtains, so as to demonstrate its potential for seclusion. Photo by coauthor Felicity Callard. (Color figure available online.)

**FIGURE 3:**
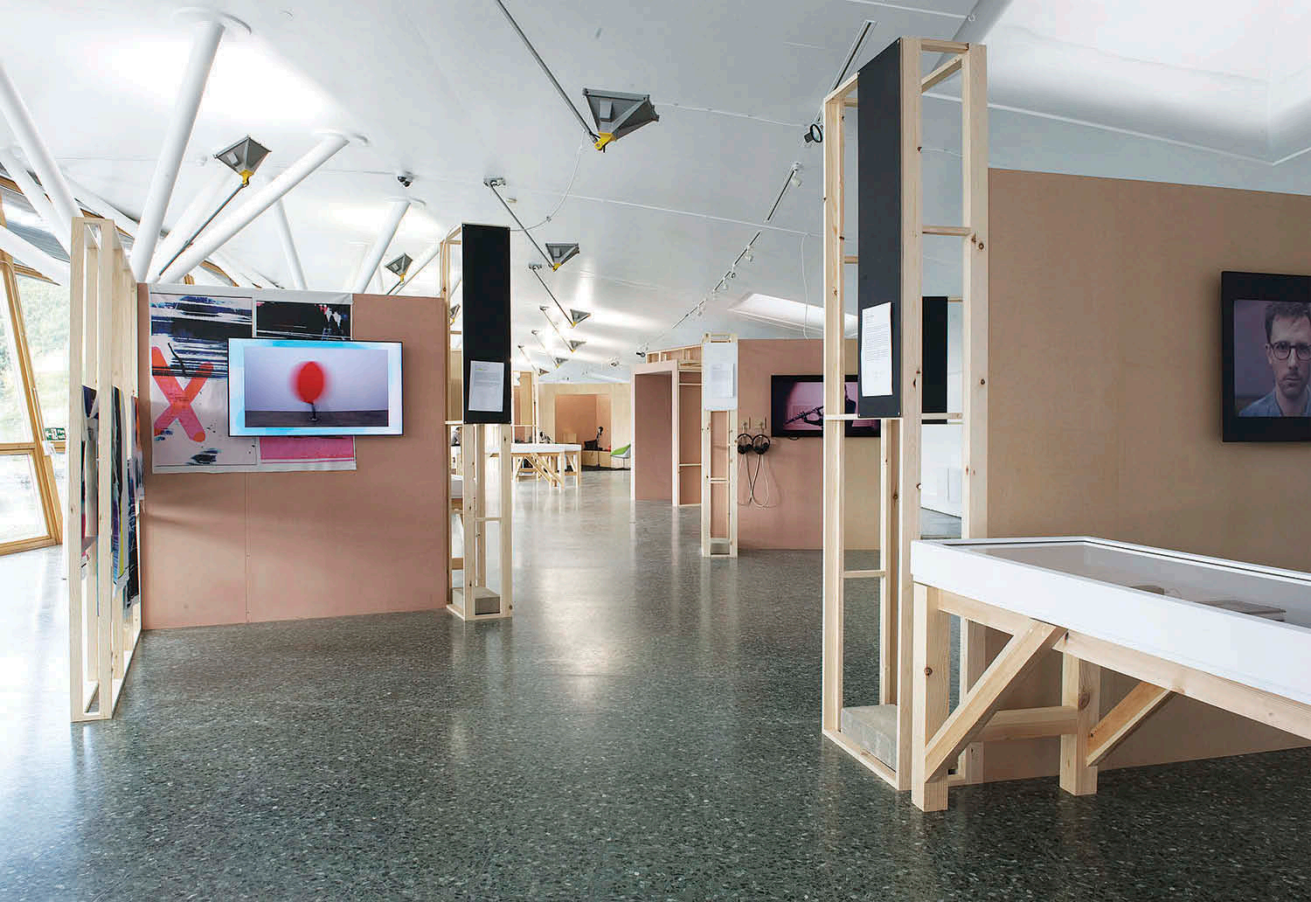
“Rest & its discontents” at Mile End Art Pavilion, London. The *Cubiculum* is the construction with the open arch, just to the right of the center of the photograph. Photo by Peter Kidd; used with permission. (Color figure available online.)

Our visitor is now looking into the interior of The *Cubiculum*, an interactive art installation designed to stage some of the problems of tracing, understanding, and analyzing mind wandering (see Figures 1 and 2). We developed it for the exhibition “Rest & its discontents,” held in 2016 at the Mile End Art Pavilion in London (see Figure 3).^1^ The *Cubiculum*, in common with a number of the other exhibition pieces, confronted and disrupted various preconceptions about “rest,” not least the assumption that it is a state characterized by inactivity or quiescence (see Farbman 2005; Immordino-Yang, Christodoulou, and Singh 2012, for other elaborations of this conjecture). For several months we had been interrogating ideas of mental “unrest,” particularly the activity and experience of mind wandering. As three humanities and social science researchers, we were keen to open up what it means to wander within the mind. Historical geographers have long been attuned to the dynamics of wandering; it is a concept that has been indispensable to research on landscape, cities, and modernity in cultural, urban, and historical geography, and elsewhere within the humanities, where attention has fallen on the spatial dynamics of experience (Parsons 2000; Pinder 2005; Wylie 2007). Mind wandering is a kind of wandering that tends to cast fewer material traces than that of physical perambulation. Although it leaves no footprints, mind wandering does have a historical geography that, we argue, might be traced, in the form of material props, spaces, and textual descriptions. Our objective, both in the design of The *Cubiculum* and this, our reflections on that creative process, is to tilt historical-geographical and cultural research toward attending as closely to the patterned, heterogeneity of so-called internal landscapes as it does to external ones. John Wylie’s now canonical cultural-geographical and formally experimental account of his “single day’s walking” on the South West Coast Path “consider[ed] again the question of how the geographies of self and landscape might be written” (Wylie 2005, 245) and, in turn, helped foment a rich and heterogeneous literature on precisely that question (e.g., Merriman and Webster 2009; Edensor and Lorimer 2015). We take inspiration here from Wylie’s and others’ efforts to address, through prose, the complexity of physical wandering and perambulation, but we do so by turning our attention inward (although this adverb will be put under pressure in the course of our article), to paths less trodden in cultural geography and in the geohumanities: namely to those traced through the mental movements of the mind.

As researchers, we focus largely on the past—we comprise a medieval historian and two geographers who work on the nineteenth and twentieth centuries. Yet, for several years we have been in dialogue with cognitive neuroscientists at the vanguard of mind-wandering experimentation and research. We decided to design a collaborative project drawing on our thorough, disciplinary analysis of archival resources as well as contemporary scientific research publications and practice, which would showcase how mind wandering has been explored, depicted, managed, and celebrated in Western literary, theological, and scientific traditions. Could such a collaboration extend beyond producing something purely pedagogic—both to afford different ontologies and epistemologies of mind wandering, and to stage some of the archival challenges associated with working on a fugitive mental phenomenon?

Our affirmative response to this question took the form of an art installation. Installation art works with, and challenges, relations between audience and visitor, art and space (González 1998). Besides detailing how mind wandering had been construed differently at various historical moments and in diverse contexts, our attention had been captured by the mechanics of its elicitation, in the practices employed by the poet, author, or scientific experimenter to induce mind wandering within themselves or their participants. We wished explicitly to consider and bring to life the ways in which moving minds have (and continue to be) constituted through and constrained by bodily, spatiotemporal, and cultural configurations (cf. Ogden 1999, on reverie in the psychoanalytic consulting room; Galison 2004, on how the Rorschach projective test unfolds and constitutes the self; and Cervenak 2014, on the potency of daydreaming and wandering for those whose mobility has been violently curtailed). An art installation offered a unique way to stage some of the complexities of mind wandering and to explore creatively the interplay between body and mind, between states of attention and inattention, and ideas of the internal and external, motion and stillness, observation and experience.

The *Cubiculum* came to function simultaneously as an art installation, an experimental situation (in which its visitors took on the role of both experimenter and experimental subject), a space of and for withdrawal and reverie, and, finally—as we indicate in the concluding section—a means of opening new directions for the historical geography of the mind. In this article, we reflect on how this project came to fruition, a journey that was, itself, a form of wandering, for, like our hypothesized visitor earlier, it was very much a step into the dark unknown.

We have elected to focus on two aspects relating to the epistemologies negotiated within the project and its creative process. First, we wanted visitors to the exhibit to be able to explore the subject heuristically, and not to be subject to a passive learning environment. With that goal in mind, we deliberately introduced into its formal design some of the spatial, bodily, and perceptual specificities used in the depiction or elicitation of mind wandering. By offering particular bodily and perceptual affordances, we sought to re-create the conditions we believed to be conducive to mind wandering. We hoped visitors might sample the experience for themselves while seated in the space. Second, we are aware of the centrality of narrative as a mode of epistemology both within the visitor experience of The *Cubiculum* and this, our retelling of the design process. Not only was the visitor invited to engage with narrative accounts of mind wandering and related phenomena: These accounts were mediated through our academic analyses—through our narratives of those narratives. Although narrative is indispensable, being the primary mechanism through which interior experience can be made known, it is equally insufficient. Mind wandering occurs outside of the grasp of attention and its ordered temporalities: It is cognizable only post facto. How much of the qualia of one’s experience of mind wandering is overlooked when that experience is squeezed into a communicable narrative?

This is one of the reasons why we did not, in writing this article, solicit the thoughts and reflections of visitors to the exhibit. We wanted to stage attempts—including ours—to elicit mind wandering, at the same time as we wanted to refuse the fantasy that that experience might ultimately be adequately captured through narratable means. Rather than cleave to the more standard social-scientific path of including visitor testimonies or feedback (e.g., M. Gallagher 2015) relating to The *Cubiculum*, we decided that the only story we had to tell was our own and that of our hypothesized visitor, the avatar of our own imagination. We have made the decision to intercut our academic register with creative extracts written in the second person, not to convey how The *Cubiculum was* experienced by the visitor but how we, the designers, imagined that it might be. As you will see, those extracts focus much more on sociospatial contexts than on the textures of our avatar’s inner life; those remain, to us, largely opaque.

## WANDERING MINDS

Whilst everyone believed that she was paying attention she was really living out fairy tales; however, she always responded immediately when addressed, so no one ever knew of this. (Josef Breuer in Hirschmüller 1989)^2^

In the last decade, there has been an efflorescence of scientific research on mind wandering (e.g., Smallwood and Schooler 2006, 2015; Christoff et al. 2016). This has come in the wake of a number of decades in which the study of such phenomena was distinctly marginalized. This efflorescence has been aided by a turn away from both behaviorist and certain cognitivist models of the mind in which attention to the phenomenological textures of consciousness was deprioritized. The study of inner mental experience is no longer rejected owing to the so-called empirical elusiveness of subjectivity (Schooler and Schreiber 2004). There is currently a lively psychological and cognitive neuroscientific field that is attempting to investigate human consciousness and phenomenology (including, centrally, “spontaneous thought”), and that is interested in the use of various introspective methodologies for accessing inner mental life (S. Gallagher 2003; Jack and Roepstorff 2003; Hurlburt and Schwitzgebel 2007).

Such research commonly defines mind wandering as the point at which an individual engages in thoughts and feelings unrelated to the task at hand (e.g., Andrews-Hanna, Smallwood, and Spreng 2014); that is, with something besides his or her immediate external environment. This definition is in part shaped by the parameters of contemporary psychological and neuroscientific experimental procedures, which have tended to study behavior and brain activity by distinguishing between “on task” and “off task” (i.e., resting, or internally oriented) periods of activity.

Alongside this scientific research runs a seam of interpretive social scientific and humanities research on how mind wandering and daydreaming have been conceptualized and represented at different historical moments (e.g., Sutton 2010; Marcus 2014; Lemov 2015; Callard 2016; Morrison 2016, forthcoming). Although earlier psychoanalytic and literary influences are now largely studied by those in the humanities and social sciences—and have largely dropped out of current scientific research paradigms—we argue that the process of revisiting earlier approaches might afford greater possibility for renewed, dynamic understanding of human consciousness, and in particular that of the wandering mind (see also Callard, Smallwood, and Margulies 2012). The *Cubiculum* was both an argument for and a product of such endeavor. We, its creators, wanted to render visible how the humanities, arts, and social sciences might, in their articulation with the life sciences, open up different epistemologies and ontologies (see also Fitzgerald and Callard 2015; Pester and Wilkes 2016). Separating on-task and off-task activity is a very particular way of understanding action, attention, and phenomenological experience in relation to mind wandering, for example, and we were keen to explore historical and archival sources that present other ways of understanding and modeling fugitive mental phenomena. Additionally, the fact that discussions of mind wandering and daydreaming extend much further back than the late nineteenth century—the historical moment to which more recent investigators tend to turn for inspiration or precedents (particularly via William James; see, e.g., Christoff et al. 2016)—offers hitherto unexplored possibilities. How might working with texts from the Middle Ages, as well as from modernity, expand and complicate a historical geography of the moving mind, and offer new conceptual resources for those from all disciplines interested in understanding how the mind interacts with both inner and outer worlds? We might draw an analogy here with Lilley’s (2011) effort to “challenge orthodox views of geography’s history” through his reconsideration of what medieval geography might do to and for our current accounts of historical geography, and through his interrogations of how “medieval geography” has been represented in histories of geography.

## INCUBATING THE *CUBICULUM*

There is a state of mind … which shows with what faculty hallucination can be produced: I speak of reverie … . Carried away by these day-dreams, these castles in the air … our thoughts expand, chimeras become realities, and all the objects of our wishes present themselves before us in visible forms. (Brierre de Boismont 1853, 42–43)

The remit we chose for ourselves was remarkably ambitious. Our inchoate thoughts during the early stages of our collaboration were the very epitome of “castles in the air”; fanciful, glorious—and impossible. We began work with rich material sources: manuscripts, sound recordings, and leather-bound books. Our initial intention was to display facsimiles of the sources. It soon became apparent, however, that this was neither financially feasible nor conceptually satisfying. Although beautiful to behold, it was their content, the narratives contained therein, that offered the window onto the cultural history of mind wandering. These were sources that needed to be heard rather than seen or read. Our journey might have started with the textual artefacts of our academic practice, but we were determined to distance ourselves from traditional modes of academic dissemination. We were not content to settle for a listening dock and a podcast: We wanted to provide our visitors with a multisensory experience.

Our plans now ran to a three-dimensional exhibit complete with surround sound; like Brierre de Boismont’s anonymous addressee earlier, we were very much in danger of being “carried away by these day-dreams.” Pragmatic concerns, however, intervened; temporal and budgetary constraints demanded we downsize our daydreams—although we would not dispense with our commitment to engendering heuristic engagement with the topic of mind wandering. The three of us had become interested in the environmental and sensory precursors to mind wandering. What were the preconditions that made such an experience likely to occur? Scholars working in and beyond the field of exhibition design, as Driver (2017) noted, “know well that space, like language, is not a neutral surface over which knowledge travels” nor an “empty container into which we can pour our learning”; rather, space “re-shapes that knowledge in significant ways” (85). We had to give careful consideration to how we designed the space so that it might encourage our visitors’ minds to wander. We consulted set designers, visual artists, and sound artists with the aim to make expressible subject matter that is in many ways intangible yet exists through the interaction of human–environment practices and performances.

We had identified archivally a series of bodily and perceptual affordances that repeatedly featured in the context of mind wandering experiences. These included solitude, physical enclosure, and a muted sensory environment, although the evidence was occasionally contradictory, particularly with regard to the last two categories. For example, in the Middle Ages, enclosed spaces that precluded both sight and sound were variously reported as both suppressing and stimulating mind wandering. In the first century CE, the Roman orator Quintilian described how the rhetorician Demosthenes “used to hide away in a place where no sound could be heard and no prospect seen, for fear that his eye might force his mind to wander” (Quintilian 2001, iv, 349). The same argument was used some 600 years later in Bede’s *Life of St Cuthbert*. When Cuthbert withdrew to the remote Northumbrian island of Inner Farne to pursue a more austere and contemplative life, he deliberately built his hermitage “so that [he] could see nothing except the sky from his dwelling, thus restraining both the lust of the eyes and of the thoughts” (Colgrave 1985, 217). Enclosure in a cramped cell with limited sensory stimulation was supposed to guard against unwelcome, wandering thoughts. Yet, physical constraint also appears to have afforded opportunities for mental extension (Carruthers 2018, 3). The twelfth-century abbot Peter of Celle spoke fondly of his monastic cell, his “room of silence,” where, he wrote:

I draw deeply from the quiet which has now been granted me. The mind has a more extensive and expansive leisure within the six surfaces of a room than it could gain outside. In fact, the smaller the place the more extended the mind, for when the body is constrained the mind takes flight. (Peter of Celle 1987, 139)

Whether preventative or productive, ideas about physical confinement and seclusion were highly implicated in medieval narratives about mind wandering.

Reflections on the importance and variety of sensory stimulation were equally conflicting. In the poem “Frost at Midnight,” a meditation on daydreaming and creative inspiration written by Samuel Taylor Coleridge in 1798, the narrator’s room is lit by the “thin blue flame” of his “low-burnt fire,” which gives off just enough light for him to see the film fluttering in the grate, reminding him of a long-forgotten memory. Later in the poem, it is the “gentle breathings” of his son that spark a flight of fancy. A series of almost imperceptible perceptual affordances sets the narrator’s mind wandering. Gentle external stimuli that soothe rather than intrude were also mentioned by the psychologist Theodate Smith who listed “[t]wilight, moonlight, solitude, … sound of the waves … watching an open fire” (T. L. Smith 1904, 467) as contexts conducive to daydreaming. Perceptual phenomena, however, need not necessarily be in motion to provoke mind wandering; static visual cues often sufficed. Virginia Woolf’s 1917 short story “A Mark on the Wall” was an attempt to document verbatim the turn of thought as the narrator mulled over the possible explanations for a mark she had spotted on the wall above the fireplace (Woolf 1919). The cognitive neurosciences, meanwhile, have consistently selected static over dynamic visual stimuli to induce mind wandering states. Participants lying in the magnetic resonance imaging (MRI) scanner are typically invited to fix their gaze on a simple quadrilateral cross.

Whether the sensory stimulation remained in stasis or in motion, the majority of the archival evidence we had collected corroborated the observation made by Singer in the 1960s that mind wandering stems from “[a] relatively monotonous external environment” (Singer 1966, 43). Moreover, it also anticipated research in the recent imaginative mobilities literature in geography: Gacek, for example, analyzed the work of the imagination in carceral spaces and drew on Edensor’s formulations in which “the melding of illumination and darkness,” gloom and solitude, are conditions that are productive of a “heightened, tactile sense of mobility” (Edensor, quoted in Gacek 2017, 73, 76). Our task (and that of our design team) lay in designing an interior space that might allow some of these historical and sociological commentaries to come to life—that would, in other words, conjure complex transactions between and across external and internal worlds.

*Inside it is dark. You blink and wait for your eyes to acclimatize. Something is being projected into the wall to your right and from the faint, flickering glow you see that the bench to your left is empty. You sit. The bench is hard. It’s fine for one but accommodating two would be tight. It’s pretty narrow. If you were to lock your hands behind your head, your elbows would touch the sides. You lean against the back wall. The darkness is a sharp contrast to the bright sunlight and white walls of the gallery. You take a deep breath and savor the moment, a welcome pause in a busy day.*

Our design aesthetic consciously coopted some of the spatial, bodily, and perceptual specificities used in the depiction or elicitation of mind wandering in the past. The booth allowed for just one visitor at a time, thereby providing a space for solitude amid the open plan layout of the gallery. The curtains not only blocked out the light and sounds of the external exhibition space, but gave the visitor complete seclusion. The lighting was deliberately muted; the only light source was a film projected onto the wall opposite the bench that ran on a loop.

Equally deliberate was how the design of the structure would reference content from the passages we had decided to play within the space itself. We had selected six sources drawn from our archival research that showcased various literary, psychiatric, psychoanalytic, and scientifically experimental engagements with or responses to the wandering mind over the past 900 years. The earliest case study was a monastic parable on wandering thoughts from the early twelfth century. While at prayer and seated inside a low, cramped cell, the protagonist of the tale, a monk named Dunstan, had allowed his mind to wander. Dropping his hands to his side, he stared out the window and watched as the devil shifted into a variety of different guises—the manifestation of his wandering thoughts (Osbern of Canterbury 1874 84–85; Eadmer of Canterbury 2006 66–68; Powell 2018). The *Cubiculum*’s physical build, its positioning of the viewer within a dark, enclosed, isolated space, was intended to make knowable the material solitude of the monk’s cell.^3^ Furthermore, in the Middle Ages the word *cella* was often used metonymically for the mind engaged in spiritual thoughts and the invention of prayers (Carruthers 1998, 85, 174). Thus, as well as being an instantiation of Dunstan’s cell, The *Cubiculum* afforded the mimetic realization of Dunstan’s experience: In stepping inside the visitor was, in effect, entering Dunstan’s headspace.

We had also imagined that the cramped quarters of The *Cubiculum* might resonate with the latest of our case studies that featured the scientific protocols used in connection with MRI machinery (the technology that contemporary cognitive neuroscientists use to map blood flow and oxygen metabolism in the brain). The MRI machine offers a not-incomparable experience to being immured in a medieval cell. The participant is placed within a narrow, confined, and solitary space with limited sensory stimulation. A standard scientific description of the MRI scanning environment characterizes it as “cramped, claustrophobic, and noisy,” noting that “the subject must lie still because movement in the scanner can produce artifactual signals” (Passingham and Rowe 2015, 29). The MRI scanner, like the medieval *cella*, involves the individual withdrawing from the social world and entering a space specifically built to investigate the workings of thought. Visitors to The *Cubiculum* effected a similar retreat; they were secluded from, yet remained tangentially present within, the gallery space.

*Cubiculum* is the Latin word for bed chamber. In the Middle Ages it was often used as a synonym for *cella* and was a place of mental rest and physical repose. Monks in the Carthusian order, for example, each had their own individual cell, but the most interior part where they slept and prayed was called the *cubiculum* (Peters 2015). Rest—and how it might be created, confronted, and disrupted—was the concept on which the whole exhibition pivoted. It was also the thread that strung our case studies together. Although there is nothing inherently restful about lying inside an MRI scanner, the other case studies present their subjects in states of physical repose: hands lying idle in the case of Dunstan; the narrators of “Frost at Midnight” and *The Mark on the Wall* both sitting comfortably in front of their fires; the opium users described by Brierre de Boismont experiencing hallucinations amid the opulence of Parisian hashish dens; and Anna O. reclining. Although The *Cubiculum* could not, primarily for budgetary reasons, realize the same degree of comfort, the notion of a bed chamber was not entirely inapt in the context of the case studies we had selected. The concept of a cubiculum as both a material and abstract space for private thought meant that it was an ideal vehicle through which to explore histories and geographies of the wandering mind.

## MOVING MINDS

*Now settled on the bench inside the dimly lit* Cubiculum, *you gaze up at the animated film being projected onto the wall opposite. You see a series of glowing lines being continually drawn and redrawn against a black background. Circles, wavy lines, and geometric shapes grow and shrink, disassemble and reform. Gradually the abstract lines morph into images: an owl, a swaddled infant, a burning cigarette. No sooner do you recognize the image than the lines once again dissolve into abstraction, a series of pulsing circles or dots bursting like fireworks. You watch as a Parisian street scene explodes into a bowl of chrysanthemums.*

**FIGURE 4:**
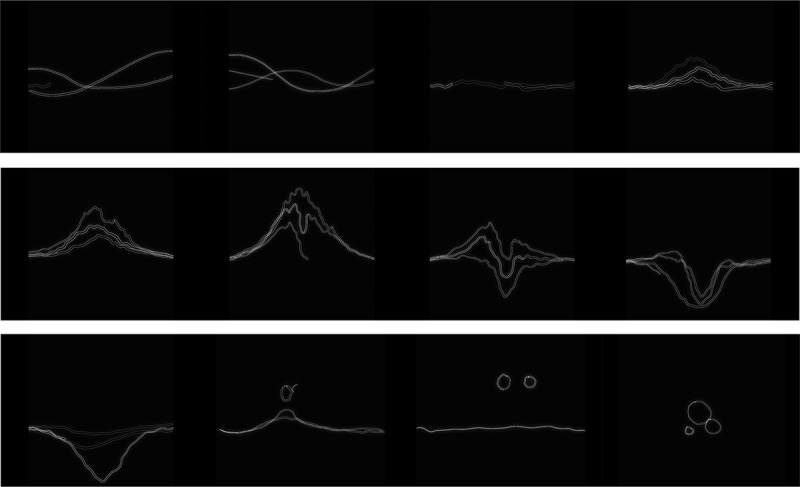
The *Cubiculum* video screen: Still image sequence from the opening animated film. Reproduced with permission of the artist, Ed Grace.

**FIGURE 5:**
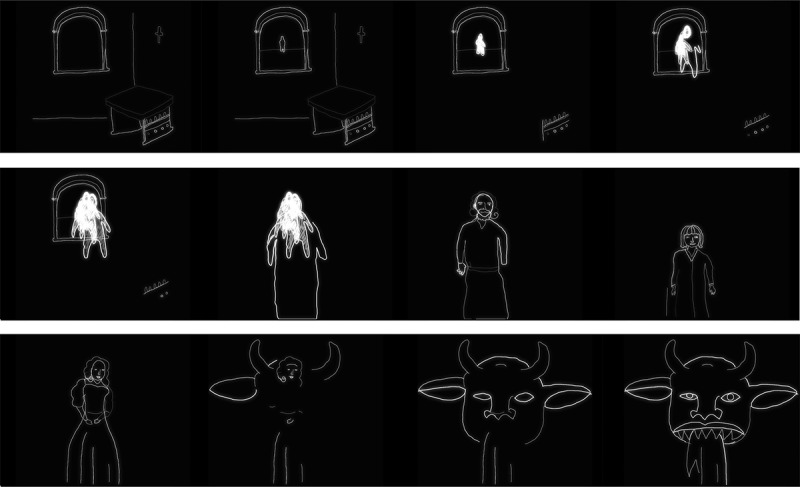
The *Cubiculum* video screen: Still image sequence from “Dunstan and the Devil.” Reproduced with permission of the artist, Ed Grace.

**FIGURE 6:**
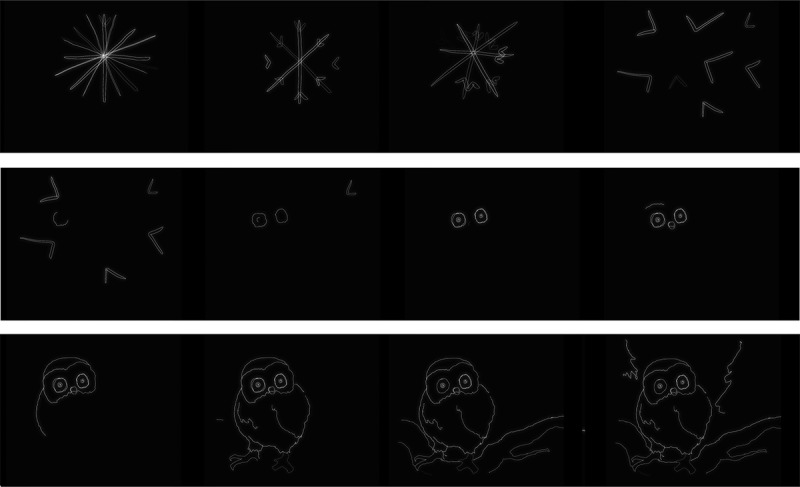
The *Cubiculum* video screen: Still image sequence from “Frost at Midnight.” Reproduced with permission of the artist, Ed Grace.

The desire to integrate some of the spatial, bodily, and perceptual triggers that we had identified into the structure of The *Cubiculum* carried into the design for the interior and content of the installation. We commissioned artist Ed Grace to produce a short, animated film containing not only the instructions for using The *Cubiculum* but his own creative response to the primary archival materials and experimental protocols that we had given him as well. The film played on a loop, offering no sense of a beginning, middle, or end that might have jolted visitors from their reverie. The glowing lines in perpetual motion had a mesmerizing effect (see Figure 4). It is not inconceivable that some visitors might have found Grace’s animation more absorbing and compulsive than their experience of any one of the case studies. Every so often the lines would come together to form a recognizable image drawn from one of the six case studies (see Figures 5 and 6). “It was extremely important” reflected Grace, “that my working method allowed for randomness, unpredictability and spontaneity, three qualities which I associate with the behaviour of my own wandering mind, but which I do not normally associate with the production of computer animation” (Ed Grace, personal communication by email, December 5, 2016). Grace made the animation using a free-drawing program called Alchemy (Willis and Hina n.d.) because it allowed him to “remove [himself] from familiar working patterns and software” and so produce an animation “truer to the free-flowing and obviously imperfect nature of mind wandering” that characterized his own free-flowing thoughts. The drawing program included a number of unusual features such as the lack of an undo function, the perpetual erasure of old marks made on the screen as new lines are drawn in, and the random allocation of color and texture. Grace wished “to create images which are vaguer than my usual output and hence more open to the viewer’s own interpretations” (Ed Grace, personal communication by email, December 5, 2016).

*As the image of the snail dissolves for the second time, you move your eyes to look at the text projected onto the wall above the surging lines. Two columns containing three items: six options in total. After a few moments you realize that these options correspond to the six silver buttons embedded in an armrest to the right of your seat. Shiny, circular, and satisfyingly tactile. Stifling the urge to press one at random, you focus again on the menu. The options strike you as esoteric—yet you feel a stab of pride when you realize that two of them relate to literary authors whose names you recognize. “The Daydreams of Anna O.” also rings a distant bell … Two others leave you intrigued: You reckon you can guess what “Hallucinations on Hashish” and “Dunstan and the Devil” might be about. The last one, however, “Modern Mind Wandering” leaves you completely baffled. Should you press to find out or play it safe and pick the poem you read at school?*

**FIGURE 7:**
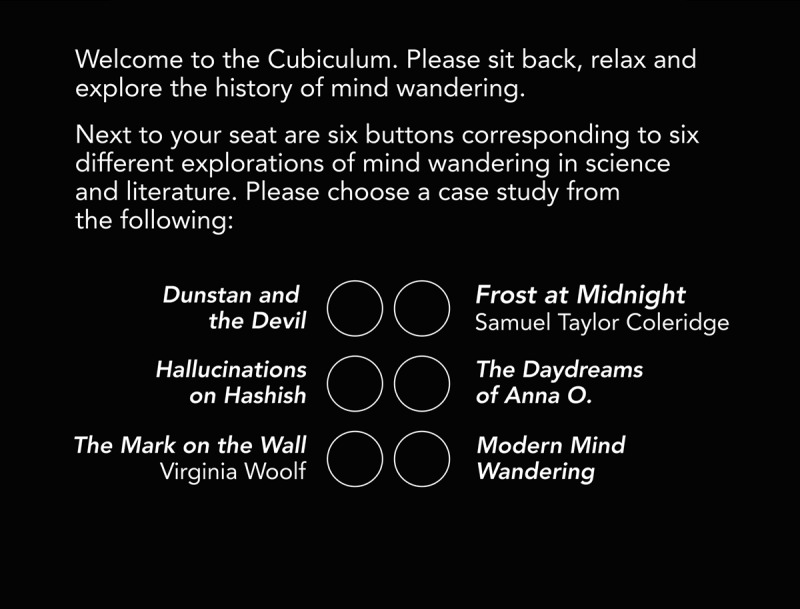
The *Cubiculum* video screen: Opening menu. Reproduced with permission of the artist, Ed Grace.

Above the animation was a menu listing the six case studies (see Figure 7).^4^ From the earliest days of the project, our aim had been to start from a place of epistemological agnosticism. We fully believed that medieval texts might yield as much about the dynamism and phenomenological specificities of a wandering mind as a scientific experiment employing the most sophisticated of today’s technologies. Moreover, in taking inspiration from recent historical-geographical enquiry into archival research methodologies, alongside those of performative and affect-based research, we looked experimentally to combine previously unrelated trajectories (Dewsbury 2010; P. Smith 2011). More specifically, we were interested in the performative interfaces between history, corporeality, language, and the sensorial; our eye was on the potential for “happenstance,” for “something new” to emerge (Aitken 2010). We were therefore keen to present the six options in such a way that would not privilege or appear to privilege one account over any others. Although the case studies were listed in chronological order, there was no intention that they should constitute an overarching narrative of the history of mind wandering. In particular, we were very conscious of the partiality of the selected sources, not least because they comprised Western texts and paradigms. We strove for interpretative and phenomenological opacity; it was important that visitors had the autonomy to navigate their own path through the exhibit, to select whichever of the six options, indeed if any, they wished to sample.

*You make your selection: “Hallucinations on Hashish.” As you had imagined, depressing the button was curiously satisfying. The glowing lines and menu vanish, and a green light floods the wall. It shimmers ever so subtly. A babble of voices starts up above you before giving way to the voice of a Frenchman.*

The press of any button initiated two simultaneous aesthetic experiences: an audio track featuring sound and spoken word, and a subtly changing lighting effect projected onto the wall facing the visitor. The audio pieces were played through loudspeakers located above the visitor’s head, enveloping them in sound. We wanted the visitor to feel immersed in the world that emerged, to be mentally transported across time and space into the cold, stone-built cell of a medieval monk or a babbling, smoke-filled Parisian hashish den.

Each case study had its own color—intended to complement rather than compete with the audio—which we, in collaboration with Grace, selected in response to the tone or imagery found in the archival material. Each color on the projection screen moved ever so slightly, and was intended to raise various interpretive possibilities for the visitor: It might have suggested clouds gently drifting across the sky (cf. Hall 1903), or wisps of smoke spiraling into the air. Color perception, wrote psychologist Stanley Hall, was brought into new connections with thought during daydreaming states. “[E]xceptional psychic structures” were, he claimed, the result of the ingenious adolescent—“sensitized to new harmonies of form and especially colour”—engaging in states of reverie (Hall 1904, 313–14). Our use of color replicated the interest in color perception and apperception that one can find in the archive addressing reverie and related states, and offered further opportunities for the visitor’s mind to wander.

Movement and shifting perceptual cues were not the only purposeful components of The *Cubiculum’s* aesthetics: Several of the case studies used the transition between archival text and contextualizing interpretation to explore further the representation and evocation of wandering thoughts. It became clear while writing the commentary for Coleridge’s “Frost at Midnight” that paraphrasing the poem’s content would not be sufficient: To engage with the poem demands exposure to its meter. We therefore resolved to include as many lines as possible, interspersed with only very brief bursts of commentary. This brought an interesting dialogical character to the audio piece and allowed the visitor to apprehend directly Coleridge’s sonorous language and its rhythms:

The inmates of my cottage, all at rest,

Have left me to that solitude which suits

Abstruser musings, save that at my side

My cradled infant slumbers peacefully.

’Tis calm indeed!—so calm that it disturbs

And vexes meditation with its strange

And extreme silentness. Sea, hill and wood,

This populous village! Sea, and hill, and wood,

With all the numberless goings-on of life,

Inaudible as dreams!

The poem soothes listeners as a lullaby might, perhaps transporting them into restful self-reflection and reverie. Jackson (2008) argued that the “action of [this] poem … seeks to explain something about the activity of the mind” (119). As such, visitors to The *Cubiculum* might have absorbed the poem’s significance in articulating the mental operations of mind wandering through their experience of the rhythm of these lines, but never quite listening to the information and argument presented in the commentary in which the poem was embedded.

*The recording ends, the screen darkens, and the glowing lines reappear. You watch the lines swirl into circles before coalescing to form the face of a man, a man with bulging eyes. You smile at the congruity between the image and what you just heard. The face breaks apart in a series of sinuous lines. Your eyes alight on the menu above. Will you choose to stay and listen to another or will you return to the world outside?*

The visitors could listen to however many or few of the case studies that they wished. Alternatively, they could simply sit and watch the drifting patterns—or close their eyes. Onlookers outside the installation, unless they listened hard, would have had no way of knowing the nature of the occupant’s experience. The *Cubiculum* not only featured case studies that communicated the intractability of grasping the daydream of another, but staged that intractability itself, by installing the incentive to mind wander at its center.^5^ Whether the visitors experienced transports of fancy in the space was entirely their own affair. We did, of course, hope that the visitors would reflect on their experience, perhaps even choose to share it with others, but did not solicit their accounts. To have done so would have undermined our desire to bring to visibility a tension found in many of the archival sources we had been studying, namely that between designing sociospatial forms appropriate for the elicitation and capture of mind wandering, on the one hand, and acknowledging the intractability of representing traces of those intimate, first-person phenomena, on the other.

## EXPERIMENTING WITH THE MIND

In this final section, we consider how a historical-geographical installation centered on “subjective materials” might help to open up questions about methods, epistemologies, and archives.

### Methods

The aesthetic and practical constraints and possibilities attached to any installation offer, we argue, new affordances through which to stage archives. Methodological approaches to archive-based research, suggest Dwyer and Davies (2009, 89) are becoming increasingly experimental, with qualitative approaches and collaborative artistic endeavors enlivening, animating, as well as making more uncertain, the material and documentary properties of the archive. Hayden Lorimer, in his examination of archive methodology, suggests that collaborative research alignments between historical-cultural geographers and practitioners in the creative and performing arts are displacing the very “idea” of the archive: its “origins … content … treatment” and the information it sustains are being “rendered more provisional, indeterminate and contestable” (Lorimer 2010, 253; see also McGeachan 2018). Indeed, many academic and practice-based researchers who fall within the broad compass of the geohumanities argue that creative practices might offer new ways both to think about and to practice our relations with the phenomena with which we are concerned (Yusoff and Gabrys 2011; Enigbokan and Patchett 2012; Pratt and Johnston 2013; Tolia-Kelly 2016). Indeed, The *Cubiculum’s* use of and interest in voice, sound, color, installation, movement, and mark-making to investigate relations, epistemologies, topologies, and affordances trace many other lines of filiation to other creative and critical-creative geographical works. (Indicative affiliations include McCormack 2004; Butler 2006; Scalway 2006; Yusoff 2007; Hawkins 2010; Cutler 2013; M. Gallagher 2015; and Yusoff 2015).

Our process undoubtedly started with textual sources—with tangible, often aesthetically rich, manuscripts, books, and journals. Given the possibilities of exhibition design, we wanted to capture something of the archive’s rich materiality. The notion of the wandering mind, however, so clearly allied to metaphors of movement—to the “ebb and flow” of mental activity—seemed contrary to encouraging a strong spectatorial logic. Reflecting on the modalities of performance-based methods, we came to think that the inclusion of facsimiles of our archival sources would work against the kind of daydream machine that we were attempting to build. Rather than dictate that the visitor’s approach be that of a spectator observing discrete artefacts, we aimed to bring to prominence the essential role of human–environment practices and performances in tracing and translating the workings of the internal world into the world outside. The *Cubiculum’s* physical build, its positioning of the viewer within a dark, enclosed, isolated space, was intended to make knowable the material solitude of the monk’s cell, the quiet of a cottage at rest, and in doing so, to momentarily bring into focus the comparative immateriality of the wandering mind. We chose to install diffuse “external” stimuli (e.g., the moving colors), rather than try to replicate the discrete, punctual, and tightly controlled external stimuli of orthodox psychological experiments that are built around “tasks.” We were interested in modulations of rhythm and mood, rather than in tightly corralling the visitor’s attention in relation to those stimuli. We conceptually and materially foregrounded and played with apparently dichotomous concepts—such as interiority and exteriority, transience and stability—to explore the potential of that which might exist, in fits and spurts, in those flashes of movement in between. The installation did not follow a mimetic logic—for example, by attempting to re-create material details that could be disinterred from our sources—but rather allowed for the imaginative condensation of analogous, although distinct, psychosocial experiences, through uniting The *Cubiculum*’s cubic form with varied audiovisual experiences that could be generated within it by different visitors in different ways. Further research and practice that explore how the formal and aesthetic specificities of an installation might represent archival traces in ways distinct from museum or library exhibits would, we suggest, likely yield benefits for historical geography.

### Epistemologies

What kind of historical geography, then, is an installation able to stage? For us, The *Cubiculum* was intended to bring to visibility a complex circuit that drew together the knowledge and experiences of the creators of a work (us), of those represented in its source material (“actual” and imagined figures with experience of mind wandering), and of those experiencing the work (the visitors). Thus, whereas the structure of The *Cubiculum* itself was solid, both the exhibit as a whole and the epistemologies that it staged were set in motion. The installation encouraged the visitor’s body to be relatively still and quiescent, yet this was in the service of encouraging—experimenting with—various kinds of mental (and indeed corporeal) movement (cf. Bissell 2009; Bissell and Fuller 2011). In some respects, our approach followed a more general interest, in historical geography, in “accessing, interpreting and evoking the embodied and affective dimensions of past practice” (J. Lorimer and Whatmore 2009, 675). Crucially, though, this was not pursued in the interests of bringing past mind wandering experiences full-bloodedly into the present; rather, we were committed to emphasizing how attempts to elicit and pin down mind wandering were and are often thwarted—whether those attempts are found in records of scientific experimentation past and present, in literary artefacts, or, moreover, in the desires of the creators of and visitors to The *Cubiculum* itself. Through attending to the sociospatial arrangements that have facilitated—either fortuitously or purposefully—a wandering mind, we find common cause with those historians, geographers, and anthropologists of science who have emphasized where—as well as how and when—phenomena emerge and come to have consistency (Livingstone 1995; Kelly and Lezaun 2017). Historical geographers have tended to focus less on immaterial, mental phenomena than material ones, and we believe that there is more to be done to uncover and analyze the complex epistemological implications of spatial arrangements in organizing regimes of observation, inner experience, and representation.

### Archives

What relation does a historical-geographical installation have to a historical archive? Does The *Cubiculum* construe mind wandering as a singular phenomenon with a narratable history and geography, and a recognizable archive? The *Cubiculum*, in a sense, left open to interpretation exactly what its primary content —its archive—was. Was it the six case studies? Was it the looping animation, which was itself inspired through (its creator’s) daydreams? Or was it the immaterial archive made up, collectively, of the daydreams and episodes of mind wandering of each visitor who crossed its threshold? Such questions have relevance beyond the specificities of The *Cubiculum*. Lemov (2009) demonstrated how, after World War II, various investigators across the human sciences attempted to collect, grapple with, and archive “‘subjective materials’—exactly those parts of human existence that elude capture” (62). If those investigators were attempting, through their strange archives and databases, to render those materials—which included daydreams—“capable of being processed, preserved and perhaps even engineered,” then our emphasis was, rather, on exploring and celebrating the strange ephemerality of subjective materials. As visitors to The *Cubiculum* were interpellated as subjects of an experimental MRI experiment, or enveloped in a monastic soundscape, The *Cubiculum* became a space resolutely opposed to the collection—capture—of the visitor’s own “subjective materials.” At the close of “Rest & its discontents,” the physical structure of The *Cubiculum* was destroyed, although the other elements (video, sound, animation) exist in spatially disaggregated form. The *Cubiculum* is now a kind of virtual exhibit, or dismembered archive. If, as the visitor sat within it, The *Cubiculum* functioned in certain respects as an experimental setting, this was an experiment in which the experimenter gathered and archived no data.

## CONCLUSION

Our article contends that props, sociospatial frames, and texts do allow mind wandering—and the historical geography of its investigation and depiction—to be traced. Our use of the term *trace* helps us center attention on the residual effects and complex dynamics of loss that subtend the often hard-to-narrate gap between a primary or phenomenological event or experience (here, the evanescent experience of mind wandering) and what runs after it (see Dubow and Steadman-Jones 2013 for a powerful argument concerning archive, narrative, and loss). We hope that our argument, and The *Cubiculum* itself, might encourage historical geographers and those in the geohumanities to experiment further with the staging of ephemeral archives and of ephemeral phenomena. This, we contend, might extend the repertoires through which we imagine, perform, and historicize the dialectic between the objective and subjective, the durable and the transient, the tractable and the intractable.

As creators of The *Cubiculum* and analysts of mind wandering, we felt ourselves to be like many of the elicitors and recorders of others’ daydreams that we have found in our archives. We—together with our methods, materials, and analyses—are placed in the difficult position of being “soul catchers,” to use the elegant formulation proposed by the historians of medicine and science Guenther and Hess (2016). We are chasing the immaterial, probing the conditions for its existence:

[T]he instruments and material culture of the modern mind and brain sciences function like soul catchers because they are grappling with the same problem: how to make the invisible visible, to capture and study that which seems fleeting and ethereal. (306)

What we attempted to create through The *Cubiculum* were not measurements, nor lasting impressions, but momentary and fleeting elicitations of phenomena. By creating a space in which viewers could variously respond to sound, image, movement, darkness, and enclosure, we began to propose a response to the dilemmas of “soul catching.” By attending carefully to The *Cubiculum*’s physical form, we simultaneously took seriously the body as the point at which our object of research might be felt (cf. Morris 2011). By engaging with histories of daydreaming, and through enacting and reproducing spatial conditions considered conducive to enabling the mind to wander (Singer 1966), The *Cubiculum* comprised an architectural contrivance that manipulated the viewer’s bodily posture, as well as the surrounding environmental stimuli and social-spatial boundaries.

It might still be unusual within geographical literatures to acknowledge the body, especially that of an unsuspecting visitor, as a tool for research dissemination (Parr 2001, 166), and yet it was precisely the “messy subjectivities” (MacKian 2010, 366)—the memories, movements, and distractions of individuals passing in and out of the chamber—through which we envisaged that the object of our research might be felt and made known. If the visitor in certain respects functioned in The *Cubiculum* as the center of “perspective, insight, reflection … agency” (Grosz 1994, xi), then the aim of The *Cubiculum’s* experimental design, and indeed, the interpolation of the second-person voice in this article, was not to grasp, or ultimately define those components of her ephemeral, inner mental experience. Rather we attempted to prioritize the “experience or experimentalism of thought” over the stability of meaning, “making it a matter of not recognizing ourselves or the things in our world, but rather of encounter with what we can’t yet determine—to what we can’t yet describe or agree upon, since we don’t yet have the words” (Rajchman 2000, 20).

In prioritizing the textures of experience over a kind of archival stability, we came to understand that that which we were exploring—daydreaming, reverie, mind wandering, fantasy—not only lacked a firm and enduring materiality, but was a diffracted and multiple, rather than singular, object. The archival sources we used are exceptional in their abilities to confer such a variety of phenomenological experience through their use of language and lyricality, and yet we recognize the artificiality of their artistry. The wandering mind, we argue, in all its richness, complexity, and everyday occurrence, cannot wholly be grasped through such firm and enduring representation. Rather, in recognizing the transience of our subject our project shares, perhaps, some of the epistemological and methodological openness of Cutler’s (2013) “Land Diagrams,” in which the “finished twinned study offers two simultaneous articulations of one image, reflecting different philosophical and methodological convictions” (114).

In drawing such parallels, the wider application of our research might be that we employ methods of questioning that attend, as Hayden Lorimer (2005) suggested, more to the question of “how life takes shape and gains expression” in such phenomena as “shared experiences, everyday routines, fleeting encounters, embodied movements” than attempt straightforward representation (84). Working in this theoretical vein, The *Cubiculum*—which drew on various methodological protocols or paradigms, including the MRI experiment and the medical case report, which have historically shaped social-scientific understandings of mind wandering—encourages reflection on the politics of meaning making (see Dewsbury 2010). Using visitors’, as well as our own, bodies, as a means to adumbrate shared experiences and physical spaces, we propose to expand our collective imaginations in understanding how so-called inner experiences are filtered and framed by the world around us, and in particular by the various ways in which complex dialectics of inner and outer, of movement and stillness, of proximity and distance are staged.

Through taking you with us in our attempt to approach the fugitive histories and geographies of mental wandering, we have tried to illuminate the richness of methods, tools, and spatial arrangements that might be used to probe and to approach the dappled landscapes of the wayward mind. We suggest that additional scholarly and aesthetic efforts might be paid to ensuring that those “internal” landscapes are understood to have as much historical-geographical and phenomenological variability and specificity as their “external” counterparts. Engaging in such a task, though, will demand reconciling oneself to attempting to catch that which has already gone. The material records—words, images, texts, and instruments—out of which The *Cubiculum* was built form retrospective attempts to describe phenomena that disappeared from their originator’s hold at the very moment at which they emerged.

## NOTES

1. “Rest & its discontents” was composed of heterogeneous works from Hubbub (the first interdisciplinary residency of The Hub at Wellcome Collection; see http://hubbubresearch.org), a project that investigated rest and its opposites in neuroscience, mental health, the arts, and the everyday. The exhibition was curated by one of Hubbub’s collaborators, Robert Devcic, and addressed the art, science, and culture of rest, stress, noise, and mind wandering. Reflections on the exhibition can be found in the review by Colley (2016).

2. The indented quotations that form the epigraphs for two sections of this article are taken from source material that we used in The *Cubiculum*’s case studies.

3. This twelfth-century story gave clear direction to the exhibit’s finished form: Dunstan’s cramped cell measured no more than four feet by two-and-a-half (Eadmer of Canterbury 2006, 66–67). Although the need to ensure disabled access dictated the width of The *Cubiculum*, it was approximately four feet in length (1.2 m; see Figures 1 and 2).

4. These comprised (1) the medieval saint St. Dunstan and his encounter with the devil; (2) Coleridge’s poem “Frost at Midnight”; (3) Brierre de Boismont’s mid-nineteenth-century discussion of hallucinations; (4) Anna O. (Josef Breuer and Sigmund Freud’s patient) and her daydreams; (5) Virginia Woolf’s short story *The Mark on the Wall*; and (6) current scientific protocols used to investigate mind wandering. We were keen to include different kinds of writing, different registers, different modes of address—as well as items that probed “normal” as well as psychopathological experiences. The audio pieces contained verbatim extracts from archival materials and current scientific protocols, which were read by voice actors; these were juxtaposed with commentaries read by the BBC broadcaster and Hubbub collaborator Claudia Hammond. The audio pieces can be found at https://soundcloud.com/hubbubsounds.

5. We acknowledge the insights of the audio producer and creator Nina Garthwaite, whose elaborations of how the radio can act as a mind wanderer’s medium was central to our development of The *Cubiculum*.
